# TXNIP regulates mitophagy in retinal Müller cells under high-glucose conditions: implications for diabetic retinopathy

**DOI:** 10.1038/cddis.2017.190

**Published:** 2017-05-11

**Authors:** Takhellambam Swornalata Devi, Mallika Somayajulu, Renu Anjan Kowluru, Lalit Pukhrambam Singh

**Affiliations:** 1 Department of Anatomy and Cell Biology, Wayne State University School of Medicine, Detroit, MI, USA; 2 Department of Ophthalmology, Wayne State University School of Medicine, Detroit, MI, USA

## Abstract

Thioredoxin-interacting protein (TXNIP) is involved in oxidative stress and apoptosis in diabetic retinopathy. However, the role of TXNIP in the removal of damaged mitochondria (MT) via mitophagy, a process of macroautophagy, remains unexplored. Here we investigate the associated cellular and molecular mechanisms underlying mitophagy in retinal cells under diabetic conditions. For this, we maintained a rat Müller cell line (rMC1) under high-glucose (25 mM, HG) or low-glucose (5.5 mM, LG) condition for 5 days. Our data reveal that HG upregulates TXNIP in the cytosol as well as in the MT. Moreover, mitochondrial oxidative stress and membrane depolarization occur under prolonged hyperglycemia leading to fragmentation. These damaged MT are targeted to lysosome for mitophagic degradation, as is evident by co-localization of mitochondrial protein COXIV, a subunit of cytochrome *c* oxidase, with autophagosome marker LC3BII and the lysosomal membrane protein LAMP2A. In addition, under HG conditions, there is an accumulation of dynamin-related fission protein Drp1 and E3 ubiquitin ligase Parkin in damaged MT, suggesting their roles in mitochondrial fragmentation and ubiquitination, respectively, which is absent in LG conditions. Subsequently, ubiquitin receptors, optineurin and p62/sequestrome 1, bind to the damaged MT and target them to LC3BII autophagosomes. Conversely, TXNIP knockout via CRISPR/Cas9 and TXNIP gRNA prevents the HG-induced mitochondrial damage and mitophagy in rMC1. Last, TXNIP level is also significantly upregulated in the diabetic rat retina *in vivo* and induces radial glial fibrillary acidic protein expression, a marker for Müller glia activation, and the formation of LC3BII puncta, which are prevented by intravitreal injection of TXNIP siRNA. Therefore, TXNIP represents a potential target for preventing ocular complications of diabetes.

Thioredoxin-interacting protein (TXNIP) has been defined as a pro-oxidative stress, pro-inflammatory and pro-apoptotic protein that is strongly induced by diabetes and high glucose (HG) in most tissues examined, including pancreatic beta and retinal cells.^1, 2^ TXNIP binds to thioredoxin (Trx) and inhibits its thiol-reducing and oxidant-scavenging activity, thereby triggering cellular oxidative stress and apoptosis.^3^ Trx1 is found in the cytosol and nucleus, whereas Trx2 is the mitochondrial isoform. TXNIP is primarily localized to the cytosol and nucleus, and during cellular stress, TXNIP migrates to mitochondria (MT) and activates cell death signaling by releasing apoptosis-signal kinase 1 from Trx2 trapping.^4^ We demonstrated previously that TXNIP upregulation induced by diabetes in the retina and by HG in retinal cells causes oxidative stress, inflammation and apoptosis.^5, 6, 7, 8^ TXNIP also causes mitochondrial dysfunction and bioenergetic deficiency in rat retinal Müller cells *in vitro* and may participate in autophagy and mitophagy.^7^ Nonetheless, the critical role of TXNIP in removing damaged or depolarized MT via macroautophagy, a process described as mitophagy, is yet to be investigated in diabetic retinopathy (DR) as well as in retinal cells in culture.

As the retina is a part of the central nervous system, the mitochondrion is critical for oxidative phosphorylation and ATP production from glucose and oxygen in the inner membrane electron transport chain (ETC). Nonetheless, the ETC also generates superoxide radicals, which can damage mitochondrial proteins, DNA and membrane lipids.^9, 10, 11^ To counter these reactive oxygen species (ROS), several anti-oxidant systems are present in the MT, including glutathione, Trx2, MnSOD and others. In spite of these protective mechanisms, mitochondrial membrane damage and depolarization occur in physiological and pathological conditions, including diabetes, and the damaged MT are segregated by fission.^12^ Mito-fission involves the cytosolic dynamin-related protein 1 (Drp1), which is a GTPase, and mitochondrial membrane-bound fission proteins, such as Fis1, which dock Drp1 onto the outer mitochondrial membrane.^13, 14^ In contrast, PINK1, which is an inner mitochondrial membrane kinase, accumulates at the outer membrane of depolarized MT and recruits the E3 ubiquitin ligase Parkin, which ubiquitinates outer membrane proteins, such as voltage-dependent anion-selective channel 1 (VDAC1) and Mfn2, as a mark for degradation of the damaged MT by mitophagy via the lysosomal degradation.^15, 16^

Macroautophagy or mitophagy is a complex catabolic process that degrades oxidatively damaged organelles and/or misfolded/aggregated proteins during starvation or oxidative stress to recycle the macromolecular or organelle components as nutrients.^15, 16^ Of the many autophagy-related proteins (ATGs), LC3BII (ATG8) is required for the nucleation and elongation of the double membrane autophagophore.^17^ LC3BI is conjugated with phosphatidylethanolamine (lipidation) to form LC3BII via a number of steps that involve ATG7 and ATG3, as well as ATG12, ATG5 and ATG16L.^17^ Initially, LC3BI exists as a pro-LC3B form and is cleaved by the cysteine protease ATG4B to form LC3BI, exposing the C-terminus glycine, which can be lipidated to form LC3BII.^18^ In addition, ATG4B also mediates the delipidation or removal of membrane-associated LC3BII from autophagophores to maintain a pool of LC3BI under basal conditions and regulates autophagy and mitophagy.^19, 20^ The delipidating activity of ATG4B is known to be inhibited by cysteine oxidation (Cys81) near its protease active site (Cys77) during oxidative stress.^19, 20^

To further proceed to the mitophagic flux, adapter proteins, such as optineurin (OPTN) and p62/sequestrome 1 (SQSTM1), which are receptors for ubiquitin-tagged proteins in damaged MT and a binding partner for LC3BII in autophagophores, recognize ubiquitinated cargos and links them to the LC3BII autophagophore to form autophagosomes.^21, 22, 23^ Autophagosomes carrying the ubiquitinated MT fuse with the lysosomal membrane via the lysosome membrane-associated protein 2A (LAMP2A) and finally constitute autolysosomes.^24, 25^ Acidic lysosomal enzymes digest proteins, lipids and DNA into their constituent molecules, which are recycled for cellular anabolic metabolism. Mitophagy initiation requires ROS/RNS signaling.^17, 18^ Therefore, we hypothesize that hyperglycemia-induced TXNIP expression and oxidative stress are involved in mitochondrial dysfunction, and mitophagy in DR and retinal cells *in vitro*. Indeed, our results show that TXNIP is significantly upregulated in the diabetic retina and correlates with Muller glia activation (enhanced glial fibrillary acidic protein (GFAP) expression) and autophagosome formation, as indicated by increased LC3BII puncta, whereas TXNIP knockdown via intravitreal siRNA reduces both GFAP and LC3BII puncta. Furthermore, HG induces mitochondrial dysfunction and mitophagy in retinal Müller cells in *in vitro* cultures, whereas TXNIP knockout by clustered regularly interspaced short palindromic repeats (CRISPR)/Cas9 and TXNIP gRNA prevents mitochondrial damage and mitophagy.

## Results

### HG induces TXNIP expression and mitochondrial localization and dysfunction in rat retinal Müller cells

**Figure 1:**
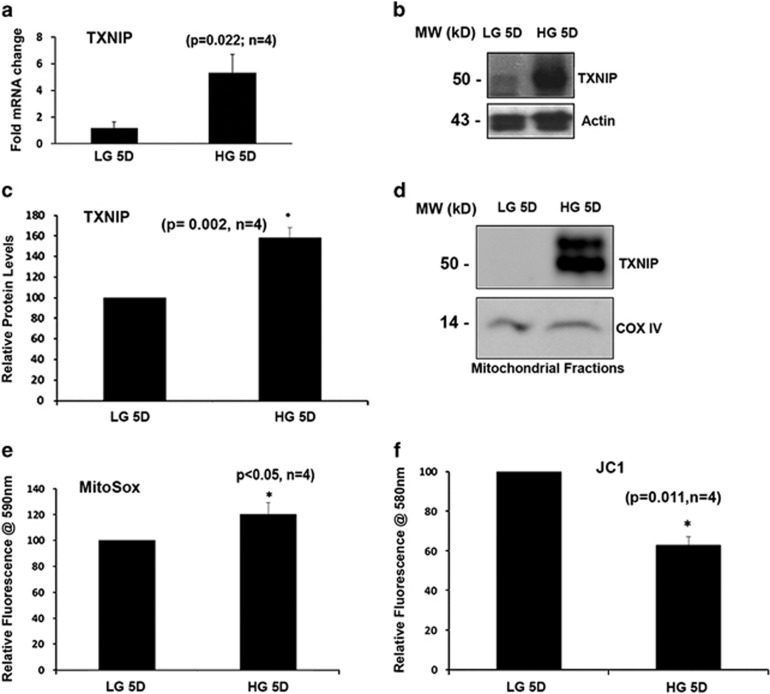
High glucose induces TXNIP expression and mitochondrial dysregulation in rMC1 in culture. rMC1 cells were maintained in LG or HG conditions for 5 days in DMEM medium containing 1% serum. (**a**) Cells were incubated in LG or HG conditions for 5 days and qPCR for TXNIP was performed (*P*=0.022, *n*=4). (**b**) Immunoblot analysis of TXNIP protein levels in rMC1 after 5 days of HG exposure. (**c**) Densitromteric quantification of TXNIP protein levels under HG and LG conditions (*P*=0.002, *n*=4). (**d**) Western blot analysis of TXNIP levels from isolated mitochondrial fractions of rMC1 under LG and HG conditions. Electron transport chain component COXIV was used as a mitochondrial marker (*n*=4). A representative blot is shown here. (**e**) Mitochondrial oxygen radical (superoxide) generation was assayed using MitoSOX (*P*<0.05, *n*=4). (**f**) Mitochondrial membrane potential was measured using the JC1 (*P*=0.011, *n*=4)

We have previously shown that sustained HG exposure causes increased expression of TXNIP and oxidative stress in retinal cells.^5, 6, 7, 8^ Here we also show that when rat retinal Müller cells (rMC1) are subjected to HG (25 mM glucose, 5 days), there is a significant increase in TXNIP expression both at mRNA and protein levels (Figures 1a–c). Interestingly, we observed increased TXNIP levels in isolated mitochondrial fractions of rMC1 cells cultured in HG (Figure 1d). Purity of the isolated mitochondrial fractions was ascertained by the absence of cytosolic protein lactate dehydrogenase and nuclear lamin B1 (Supplementary Figure S1). To confirm the presence of TXNIP in the MT, we co-stained rMC1 cells via immunofluorescence for TXNIP and Trx2. Trx2 is present in the MT and is important in combating oxidative stress.^4^ There is clear evidence of co-localization of TXNIP with Trx2 in rMC1 under HG conditions (Supplementary Figure S2).

Also, we observed that HG increases mitochondrial ROS levels in rMC1, as shown by the MitoSox assay (Figure 1e). This increase in ROS in the MT correlates with reduced mitochondrial membrane potential (Δψm↓), which is evident by a reduction in red fluorescent in JC1 assays (Figure 1f).

### HG induces mitophagy in rMC1

To understand the mechanism underlying mitochondrial dysfunction and TXNIP-mediated mitophagy, if any, we measured proteins involved in mitophagic processes in rMC1 exposed to HG. As mentioned before, mitophagy is a form of macroautophagy wherein a selective degradation of damaged MT occurs via a double membrane autophagosome. Ubiquitinated proteins on the outer mitochondrial membrane of damaged MT are recognized by adapter molecules or receptors, such as OPTN, p62/QSTM1 and so on, which are responsible for recruiting these damaged organelles to the autophagophore via their interaction with LC3BII for engulfment. These autophagosomes then fuse with the lysosome forming the autolysosome through which the degradation of the mitochondrial cargo occurs. We have used western blotting and immunofluorescence to study the involvement of these proteins in mitophagy. Initially as controls for mitophagy induction, we used carbonyl cyanide 3-chlorophenylhydrazone, a mitochondrial membrane ionophore, which induces mitochondrial membrane depolarization and mitophagy and also Bafilomycin A, an inhibitor of lysosome fusion. These results are shown in Supplementary Figure S3E.

**Figure 2:**
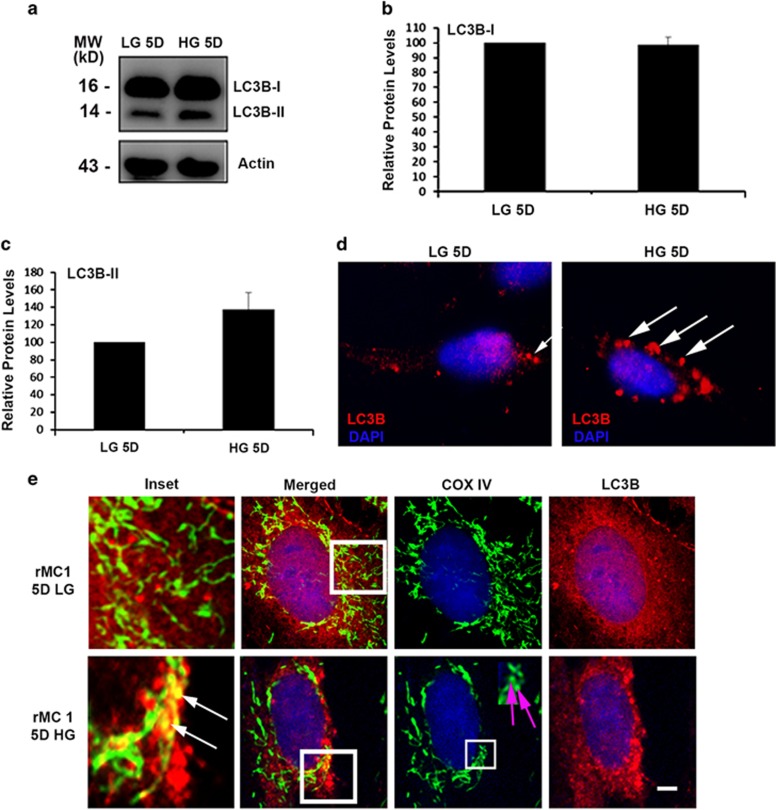
High glucose mediates macroautophagy/mitophagy induction in rMC1. (**a**) Western blot was used to measure the levels of LC3BI and LC3BII. Lipidated LC3BI or LC3BII moves faster in polyacrylamide gels than LC3BI, despite being larger in size (probably due to hydrophobicity caused by lipid conjugation); therefore, the levels of the two LC3B species can be quantitated. **(b**,**c)** Quantification of LC3BI and LC3BII levels in rMC1. All proteins are normalized to the corresponding actin level. (**d**) rMC1 cells were treated with LG and HG and immunostained for LC3B, which detects LC3BII in autophagosomes. HG-treated cells had increased LC3BII puncta as compared to LG-treated cells shown by white arrows. (**e)** Immunofluorescence studies showing co-localization between MT and autophagosome marker LC3B in HG-treated cells as shown by white arrows in insets. Also, fragmented MT are depicted with pink arrows in the COXIV column under HG conditions. Scale bar is 5 *μ*m. All experiments were performed thrice

**Figure 3:**
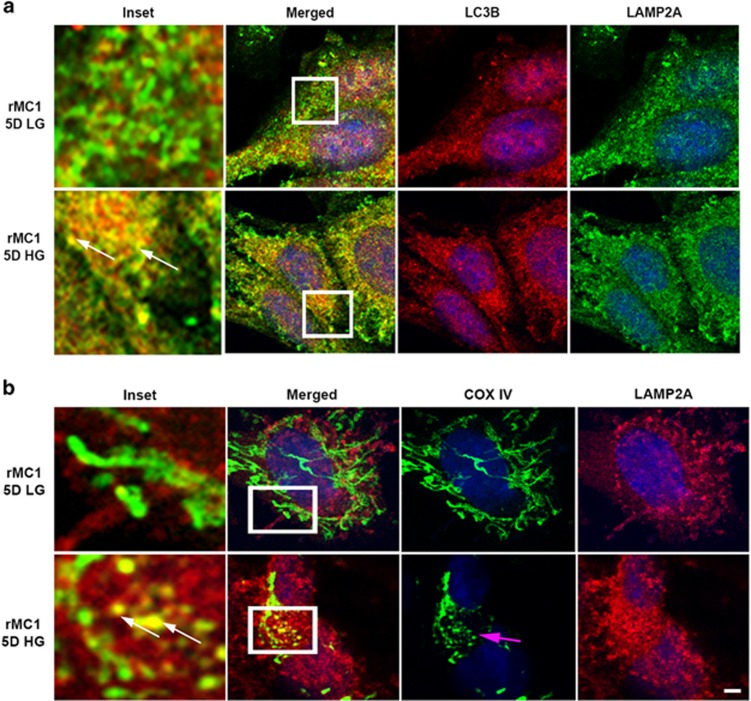
High glucose induces damaged MT flux to lysosomes in rMC1. (**a)** Immunofluorescence studies showing co-localization between autophagosme (LC3BII puncta) and lysosomal protein LAMP2A in HG-treated cells, which is absent in LG, indicating further fusion of autophagosomes with lysosomes (indicated by white arrows in inset). **(b)** Immunofluorescence studies showing co-localization between mitochondrial COXIV and lysosomal LAMP2A in HG-treated rMC1 cells, indicating fusion of damaged MT with lysosomes (indicated by white arrows in inset). Also, fragmented MT are depicted with pink arrows in the COXIV column under HG conditions when compared to LG conditions. Scale bar is 5 *μ*m. All experiments were performed thrice

In examining LC3BI and L3BII (active form) levels on western blots, we observed a marginal increase in LC3BII levels, although not significant, and no change in LC3B-I levels between low-glucose (LG)- and HG-treated cells (Figures 2a–c). However, LC3BII puncta are increased under HG conditions (Figure 2d) when compared to LG conditions. Confocal microscopy data also reveal fragmentation of MT and co-localization of the mitochondrial ETC complex protein COXIV (cytochrome *c* oxidase, subunit IV, a mitochondrial marker) with LC3BII under HG conditions (Figure 2e). Furthermore, MT are also seen fragmented under HG conditions when compared to LG conditions (Figure 2e). We further determine autophagosome flux to the lysosome. We demonstrate co-localization between LC3BII and LAMP2A (lysosome associated membrane protein 2, a lysosomal marker) in HG-treated rMC1 cells and establish that autophagosomes are fused with lysosomes for degradation (Figure 3a). To confirm that damaged MT are indeed fluxed to lysosomes, we co-stained rMC1 cells with COXIV and LAMP2A. Our data reveal co-localization of these damaged MT with the lysosome (Figure 3b), indicating mitophagy.

### TXNIP knockout prevents mitophagy in rMC1 under HG conditions

Treatment of rMC1 cells with azaserine (2 *μ*M), which inhibits the hexosamine biosynthesis pathway and TXNIP,^6^ led to the abrogation of MT and autophagosome co-localization under HG conditions (data not shown), and suggested a role for TXNIP in mitophagy. Therefore, to ascertain the role of TXNIP in mitophagy, we developed a CRISPR/Cas9 and TXNIP gRNA method to knockout TXNIP in rMC1 and examined its effect on mitophagy. Two TXNIP gRNAs (TXNIP1 and TXNIP3) targeting the rat TXNIP EXON 1 were co-transfected with Cas9 mRNA and determined the reduction in the expression of TXNIP. Co-transfection of TXNIP gRNA1+3 significantly reduces TXNIP expression in TXNIP knockout T(1+3) cells when compared with control rMC1, transfected with gRNA alone (Supplementary Figure S4).

**Figure 4:**
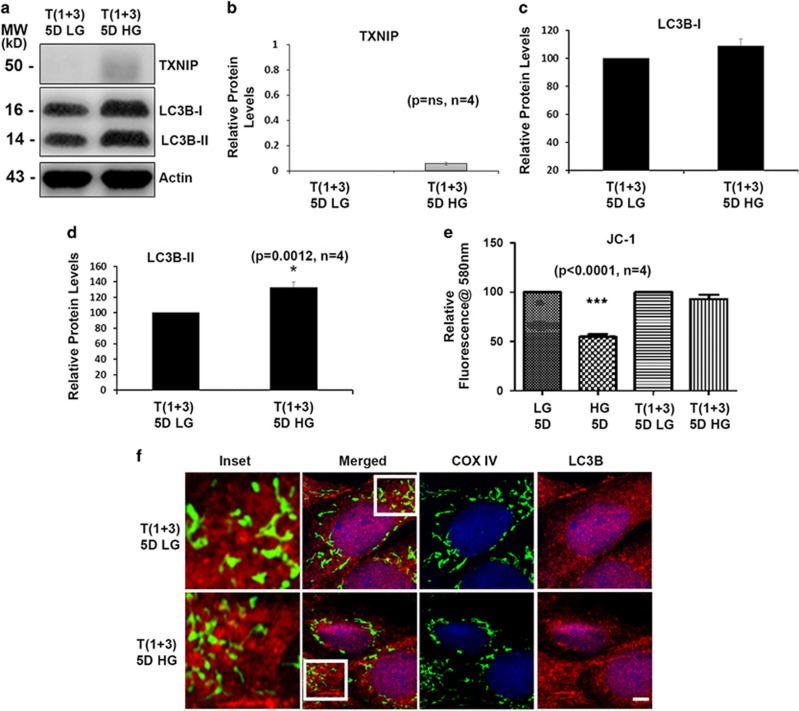
TXNIP knockout prevents mitophagy in rMC1 under high-glucose conditions. (**a**) Immunoblot analysis showing significant knockout of TXNIP levels after CRISPR/Cas9 and gRNA TXNIP treatment. Cas9/TXNIP gRNAs prevent HG-induced TXNIP expression and enhance LC3BII levels in T(1+3) cells. (**b**) Quantification of TXNIP levels after TXNIP knockout (*P*=NS, *n*=4). (**c**,**d**) Quantification of LC3BI (*P*=NS, *n*=4) and LC3BII (*P*=0.0012, *n*=4) in T(1+3) cells under LG and HG conditions after 5-day treatment. Although LC3BI remained unchanged, L3BII levels were significantly increased. (**e**) HG does not induce mitochondrial membrane depolarization in T(1+3) cells, as measured by the JC1 (*P*<0.0001, *n*=4). (**f**) Immunofluorescence images of mitochondrial COXIV and LC3B in T(1+3) cells. There is no co-localization of MT with LC3B autophagosome in TXNIP knockout cells both under LG and HG conditions. A representative image of *n*=3 is shown. The bar in the image represents 5 *μ*m

**Figure 5:**
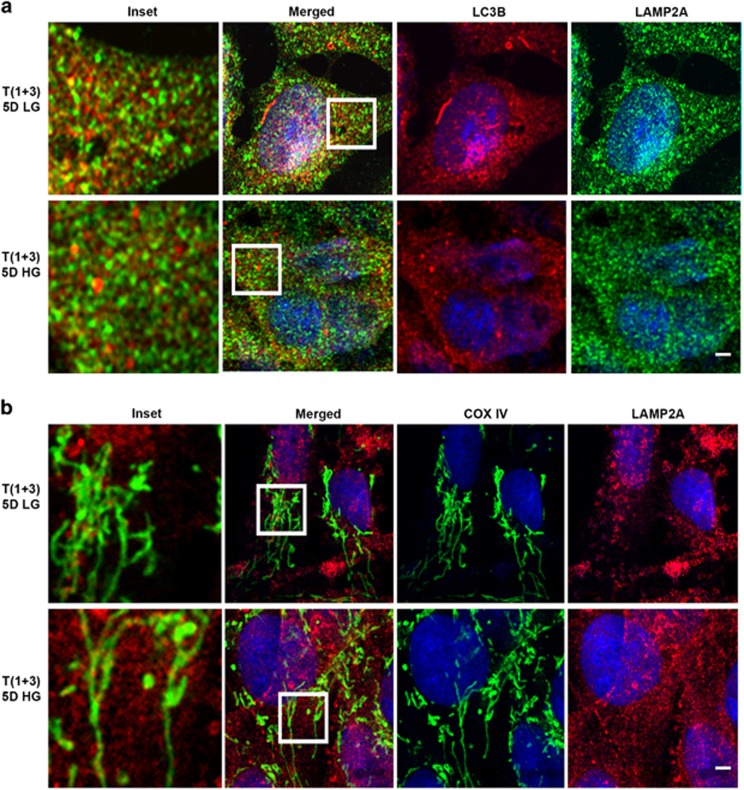
Mitophagy is reduced in T(1+3) rMC1 cells under high-glucose conditions. (**a**) Co-localization analysis of lysosomal protein LAMP2A and autophagosome (LC3BII) was performed after treating T(1+3) cells under LG or HG conditions for 5 days. No co-localization of LAMP2A and LC3BII is observed in the TXNIP knockout cells. (**b**) Similarly, immunofluorescence imaging of LAMP2A and mitochondrial COXIV in T(1+3) cells shows no co-localization between these protein either in LG or HG condition. Furthermore, elongated MT are seen both under LG and HG conditions (COXIV, green staining), suggesting that mitochondrial fragmentation is also prevented. A representative image of *n*=(2–3) is shown. The bar in the image represents 5 *μ*m

We show in Figures 4a and b that TXNIP levels are significantly lower in TXNIP knockout rMC1 under HG conditions. The level of LC3BII in HG conditions is higher in TXNIP gRNA-transfected T(1+3) cells (Figures 4a, c and d) than in LG conditions. Although LC3BI is marginally increased, it does not reach a significant value. Moreover, T(1+3) cells maintain mitochondrial membrane potential (Δψm) under both LG and HG conditions (Figure 4e). Furthermore, immunofluorescence study reveals no co-localization of mitochondrial COXIV with LC3BII (Figure 4f) in TXNIP knockout T(1+3) cells both in LG and HG conditions. We also did not observe co-staining of LC3BII and LAMP2A (Figure 5a) in T(1+3) cells in LG or HG conditions. In addition, co-localization of COXIV and LAMP2A is also absent in TXNIP knockout cells under HG conditions (Figure 5b), and the mitochondrial morphology shows elongated MT in T(1+3) cells under both LG and HG conditions (Figure 5b, COXIV staining for MT).

**Figure 6:**
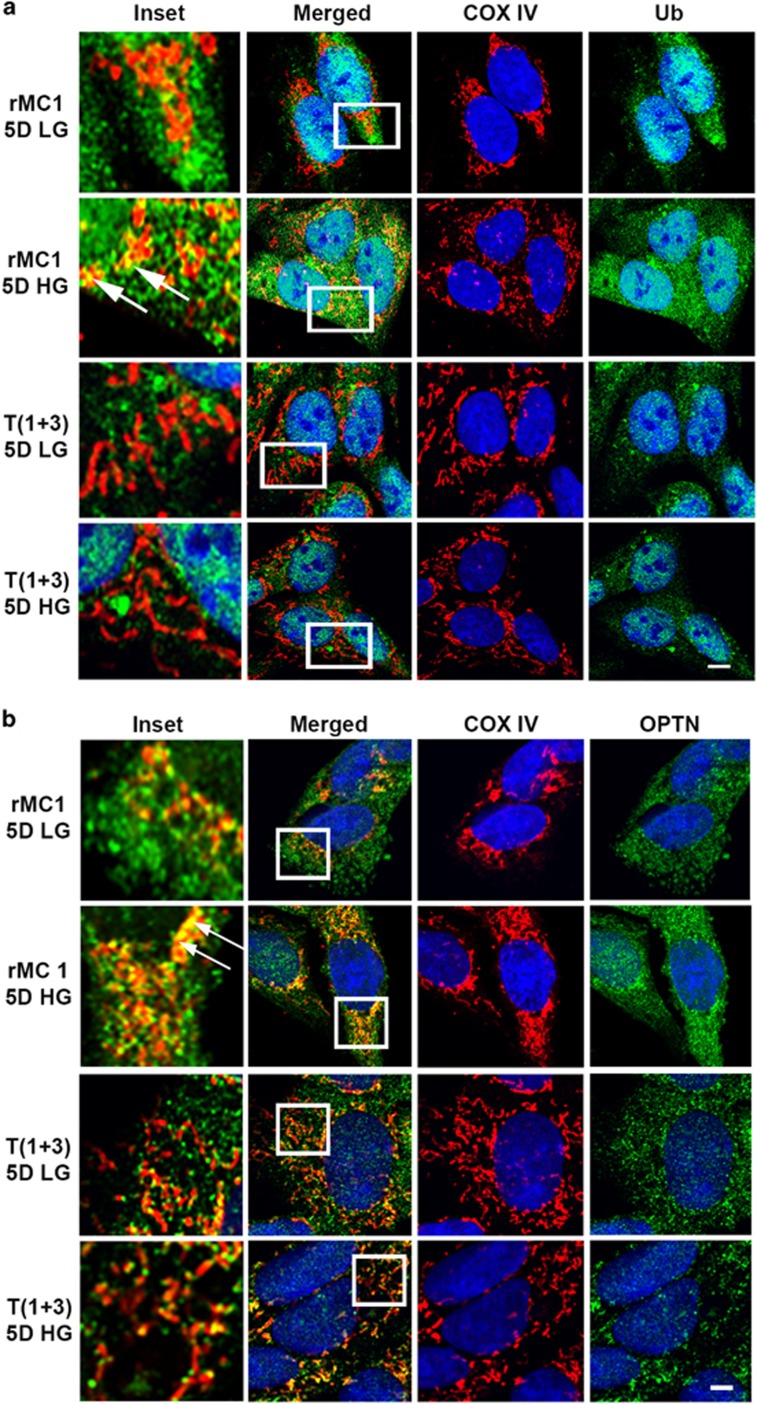
High glucose induces ubiquitination of mitochondrial membrane protein in rMC1, but not in T(1+3) cells. (**a**) Immunofluorescence showing an increased staining of ubiquitinated proteins to MT (as detected by anti-Ub antibodies) and co-localization with COXIV (boxed area in merged image and white arrows in inset). Abrogation of ubiquitin antibody binding to MT (stained for COXIV) is observed in TXNIP knockout (T(1+3) cells) under LG and HG conditions. (**b**) Immunofluorescence image showing increased co-localization of optinuerin (OPTN, ubiquitin adapter involved in mitophagy) with MT (COXIV) in rMC1 cells, which is reduced in TXNIP knockout T(1+3) cells under HG conditions. The bar in the image represents 5 *μ*m. A representative of *n*=3

As mentioned before, mitophagy is a form of macroautophagy, where ubiquitinated proteins on the outer mitochondrial membrane are recognized by ubiquitin adapters such as OPTN and p62/SQSTM1. OPTN recruits damaged MT to autophagosome via LC3BII. Fusion with the lysosome and formation of the autolysosome cause the degradation of the mitochondrial cargo. Using immunofluorescence, we show in rMC1 cells that under HG conditions, there is a higher co-localization of mitochondrial COXIV with both ubiquitin (Figure 6a) and OPTN (Figure 6b). However, when TXNIP is knocked out in T(1+3) cells, there is clear evidence that no co-localization occurs between mitochondrial COXIV and ubiquitin or with OPTN under HG conditions. Not only OPTN, p62/SQSTM1 also is associated with COXIV in rMC1, but not in TXNIP knockout T(1+3) cells (Supplementary Figure S5). p62/SQSTM1 level is also reduced under HG conditions in rMC1 cells, as an indication of lysosomal degradation, but not in TXNIP knockout T(1+3) cells. Taken together, these results demonstrate that TXNIP is involved in regulating LC3BII autophagosome in rMC1 under HG conditions and targeting damaged MT to lysosomes in mitophagy.

### A potential mechanism for TXNIP-mediated mitophagy in rMC1

**Figure 7:**
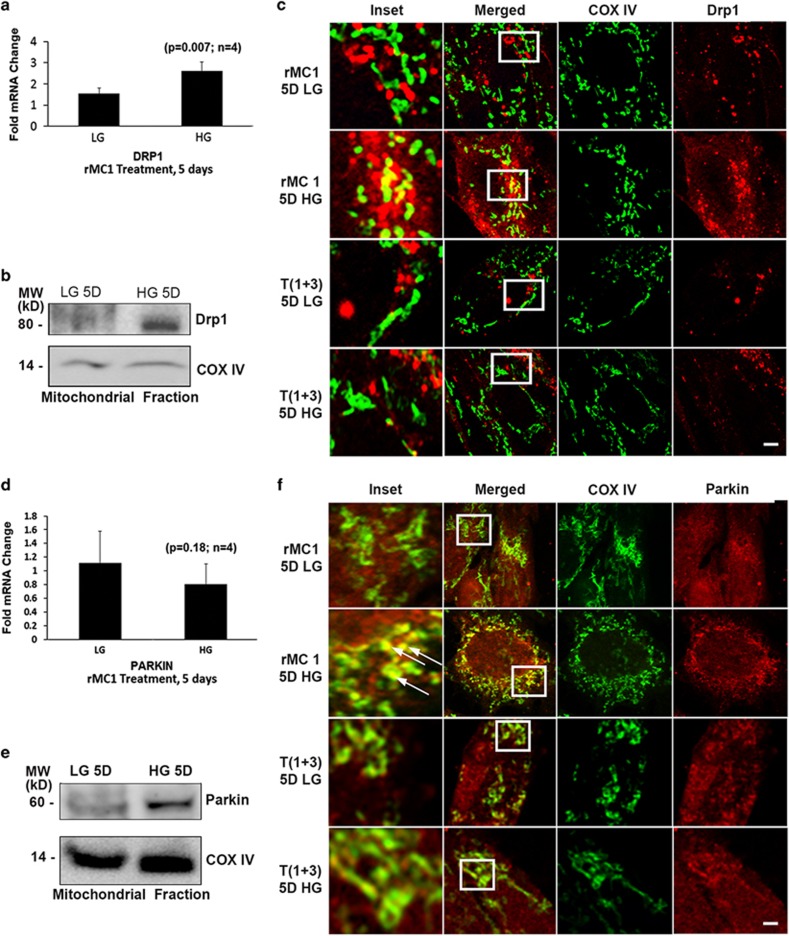
High glucose increases localization of fission protein Drp1 and E3 ubiquitin ligase Parkin in mitochondria in rMC1 cells, but not in TXNIP knockout T(1+3) cells. (**a**) qPCR analysis of mRNA levels of Drp1 in rMC1 treated with HG for 5 days (*P*=0.007, *n*=4). (**b**) Western blot analysis of Drp1 levels in the isolated mitochondrial fractions of rMC1 after 5 days of HG. A blot of *n*=3 is presented here. (**c**) Analysis of Drp1 localization in MT by confocal microscopy of rMC1 and T(1+3) treated with LG and HG for 5 days. The cells were immunostained for Drp1 and COXIV. The association of MT (COXIV, red) with Drp1 (green) is seen in rMC1 cells under HG conditions (merged, yellow in upper two panels). However, upon TXNIP knockout in T(1+3), COXIV and Drp1 stain separately under both HG and LG conditions, and no co-localization is observed (lower two panels). (**d**) qPCR analysis of Parkin mRNA in rMC1 after 5 days of LG and HG treatment. (**e**) Immunoblot analysis of E3 ubiquitin ligase Parkin levels in isolated mitochondrial fractions of rMC1 under HG conditions. A representative *n*=3 is shown. (**f**) Analysis of mitophagy by using confocal microscopy of rMC1 (upper two panels) and TXNIP knockout T(1+3) cells (lower two panels) after 5 days of LG and HG treatment. Cells were immunostained for Parkin and MT (COXIV). A representative of *n*=3 is shown for all immunofluorescence images. Scale bar represents 5 *μ*m

To further understand the potential mechanism(s) for mitophagy induction and regulation in rMC1 under HG conditions and the role played by TXNIP, we determined the expression of proteins that are considered to be key factors in MT fission and ubiquitin marking of damaged MT for mitophagy, such as Drp1 and Parkin, respectively. Mitophagy is the only mechanism for removing the damaged MT. However, the size of a mitochondrion is ~5 *μ*m, whereas that of the autophagosome is <1 *μ*m.^26^ Therefore, mito-fission to generate ~100–500 nm sizes is a pre-requisite for mitophagy. Hence, we determined whether Drp1 is involved in mito-fission in rMC1 under HG conditions. We hypothesized that if HG and TXNIP play a role in mitochondrial fragmentation by Drp1, then the cytosolic Drp1 should migrate to MT in rMC1 cells, but not in TXNIP knockout T(1+3) cells. Data reveal an increase in Drp1 expression in rMC1 under HG conditions both at mRNA and protein levels (Figures 7a–c). Furthermore, co-localization between mitochondrial COXIV and Drp1 is seen in rMC1 under HG conditions but not under LG conditions (Figure 7c, upper two panels, inset). However, when TXNIP is knocked out in T (1+3) cells, Drp1 co-localization with MT is absent, both under LG and HG conditions (Figure 7c, lower two panels).

Next, we studied the association of Parkin with MT in mediating ubiquitination. Results indicate an increased expression of Parkin protein in isolated MT of HG-treated cells (Figure 7e), although mRNA levels remain unchanged. Also, mitochondrial COXIV co-localizes with Parkin under HG conditions (Figure 7f, upper two panels). When TXNIP is knocked out in T (1+3) cells, there is no co-localization of Parkin with mitochondrial COXIV (Figure 7e, lower two panels).

It has also been shown that TXNIP and ROS stress are involved in ATGF4B inactivation, which reduces LC3BII delipidation and, therefore, enhances LC3BII-mediated autophagome formation.^18^ We observed that ATGF4B mRNA level is reduced in rMC1 under HG conditions (Supplementary Figure S6A) and also in immunostaining (Supplementary Figure S6B), which is reversed in T(1+3) cells (Supplementary Figure S6B, lower panel).

These findings together indicate that TXNIP is involved in mitochondrial fission, parkin-dependent ubiquitination, and LC3BII-mediated autophagosome formation and mitophagic flux to lysosomes in retinal Müller glial cells under sustained hyperglycemia.

### TXNIP expression is strongly induced in the diabetic rat retina and participates in LC3BII puncta formation

Further, we observed that diabetes increases TXNIP expression significantly (*P*<0.007) both in message and protein levels in the rat retina (Supplementary Figures S7A–D) when compared with the non-diabetic rat retina. Furthermore, this TXNIP upregulation correlates with LC3BII puncta formation (Supplementary Figure S7E) in diabetic retinas and also with the increases in the radial GFAP expression, indicating Müller glia activation, gliosis (Supplementary Figure S7F). Intravitreal siRNA delivery targeting TXNIP reduces TXNIP expression, LC3BII puncta and GFAP expression in diabetic rat retinas (Supplementary Figures S7 D–F), which is more or less comparable to that observed for non-diabetic rats. Furthermore, diabetes alters the expression of several genes involved in mitochondrial stress (including mitochondrial membrane pore protein VDAC1, aconitase 2 and heme oxygenase-1) and neuronal injury (such as tyrosine hydroxylase and synaptic protein synaptopodin) (Supplementary Figure S8). In addition, fission protein fis1 is significantly increased, whereas mitofusion protein Mfn2 is not (data not shown). Therefore, the data presented here both for *in vitro* and *in vivo* experiments suggest a role for TXNIP in mitochondrial dysfunction and mitophagy in the early DR. Detailed studies for an *in vivo* role of TXNIP in mitophagy are warranted and we are currently undertaking this study.

## Discussion

**Figure 8:**
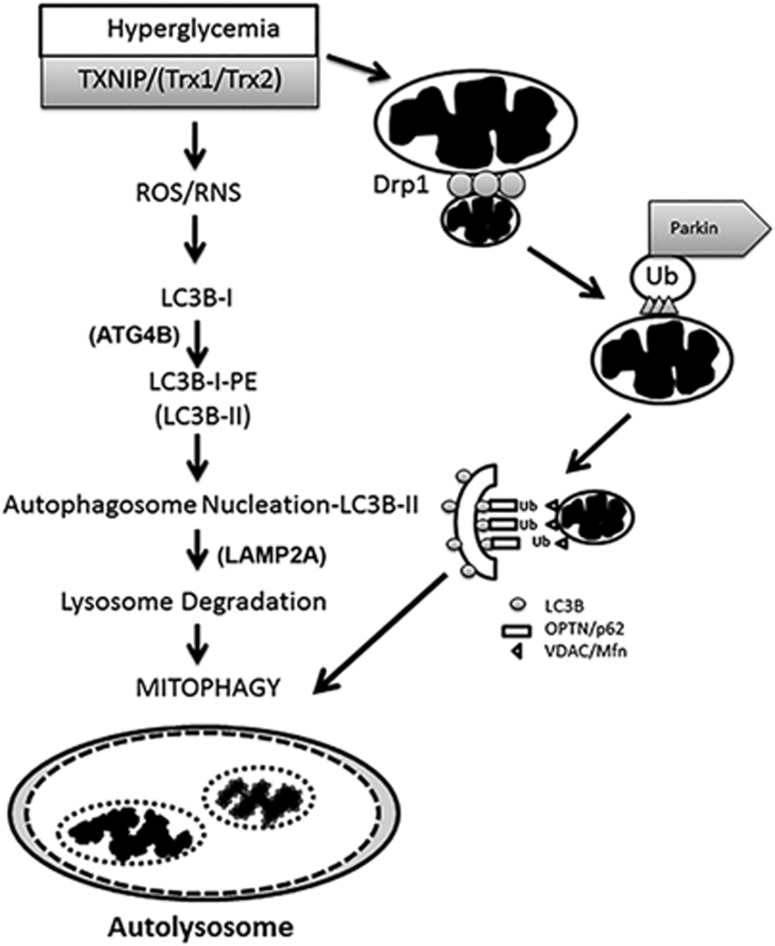
Potential mechanisms for TXNIP-mediated mitophagy in retinal Müller cells under high-glucose environment. TXNIP is strongly induced in the diabetic retina *in vivo* and by high glucose in retinal Müller cells in *in vitro* cultures. TXNIP causes ROS/RNS stress and mitochondrial dysfunction in rMC1 under HG conditions. TXNIP and ROS/RNS cause mitochondrial damage and fragmentation through Drp1-mediated MT fission. The E3 ubiquitin ligase Parkin mediates ubiquitination of mitochondrial membrane proteins, such as VDAC1 and fusion protein Mfn2. Then, ubiquitin receptors, including OPTN and p62/SQSTRM1, bind to and target the damaged MT to LC3BII autophagophores. Subsequently, lysosome and autophagosome fuse via lysosomal outer membrane protein, LAMP2A and SNARE proteins.^40, 41^ The autophagosome cargos (damaged MT and aggregated proteins) are subsequently degraded to their molecular components by lysosomal acid hydrolases. The breakdown products are recycled and reused in cellular anabolic processes. When TXNIP is knocked out by CRISPR/Cas9/gRNA, there is increased delipidation of LC3BII from autophagophores, probably via enhanced ATG4B expression and activation, which limits autophagosome formation. Similarly, mitochondrial depolarization, fission and mitophagy are reduced, and the mitochondrion maintains an elongated morphology. Thus, reducing TXNIP upregulation in DR via CRISPR/Cas9 and TXNIP gRNA may be one approach for long-term gene therapy to prevent or slow the progression of diabetic ocular complications

The results of the present study provide evidence that (i) TXNIP upregulation in retinal Müller cells in *in vitro* cultures causes mitochondrial superoxide production and mitochondrial dysfunction. (ii) TXNIP is responsible for mitochondrial fission under sustained HG conditions, as Drp1 association with MT is prevented by TXNIP knockout. (iii) The association of Parkin, E3 ubiquitin ligase, is also increased in MT under HG conditions, suggesting mitochondrial membrane protein ubiquitination and recognition by adapter proteins, such as OPTN and p62/SQSTM1, under sustained hyperglycemia. These events are prevented by TXNIP knockout. (iv) HG and TXNIP alter ATG4B level and LC3BII-mediated autophagosome formation. A representative illustration of the mitophagic process in rMC1 under HG conditions is depicted in Figure 8. We also demonstrate here the feasibility of knocking out TXNIP via CRISPR/Cas9 and TXNIP gRNA in Müller cells in culture. Whether CRISPR/Cas9 and TXNIP gRNA exert off-target effects on genomic DNA in this study or in other CRISPR/Cas9 studies will not be known unless we perform complete genome sequencing. Nonetheless, we have not observed any effect of CRISPR/Cas9 and TXNIP gRNA on other molecules involved in this study.

The retina consumes a large amount of glucose and oxygen to generate ATP for its phototransducing and visual functions; therefore, efficient oxidative phosphorylation via its mitochondrial ETC is critical.^11, 12, 13^ As such, ETC complexes I, II and III generate ROS through electron leakage and interactions with molecular oxygen. Mitochondrial quality control is therefore required at both protein and organelle levels.^14, 26, 27, 28^ Mitochondrial organelle quality control involves fission, fusion, mitophagy, biogenesis and transport.^17, 18^ The fission and fusion processes are important for mitochondrial material mixing and the separation of depolarized or damaged MT for degradation by mitophagy. During hyperglycemia and oxidative stress, mitochondrial fission appears to be dominant, as the expression of Drp1 is increased (Figures 7a–c). However, fusion also involves outer and inner membrane fusion proteins, such as fis1, Mfn2 and OPA1 (optic atrophy 1).^14, 29^ Fragmented MT are inefficient in ATP synthesis, but produce more ROS, leading to mitochondrial damage and depolarization, and they are targeted for removal by mitophagy. On the other hand, we have previously demonstrated that mitochondrial biogenesis is dysregulated in DR, and the mitochondrial DNA copy number is reduced.^11, 12, 13^ What is most interesting here is that damaged MT recruited Parkin and underwent Parkin-mediated mitophagy under HG conditions (Figures 7d–f). Recent work with human retinal pigment epithelium (hRPE) has shown that with cells grown in high-glucose conditions shifted to glycolytic ATP production and away from a metabolic need on OXPHOS. Under these conditions, cells underwent Parkin-mediated mitophagy.^30^ We also observed both in human retinal pigment epithelial cell line (ARPE-19) and primary human HRPE cells that HG induces TXNIP expression and causes mitochondrial dysfunction and mitophagy (unpublished data).

The question of whether mitophagy is good or bad in neurodegenerative diseases and chronic nutrient excess, such as diabetes and its complications, has remained unanswered. Mitophagy is a survival process during stress. However, too much mitophagic flux may be undesirable. One possibility is that during chronic hyperglycemia, sustained TXNIP expression and ROS/RNS stress prevail under mitochondrial damage, which may cause a reduction of the delipidating activity of ATG4B on LC3BII. With respect to ATG4B, both a reduction of expression (this study) and oxidative inhibition^18, 19, 20^ lead to excess LC3BII accumulation and lysosomal flux depleting p62/SQSTM1 (Supplementary Figure S3) and other ubiquitin adapters. Therefore, the level of LC3BII in mitophagy could vary depending on both the activity of ATG4B and the magnitude of autophagosome flux to lysosome. A recent report suggested that TXNIP interacts with REDD1 (regulated in development and DNA damage responses 1) and induces cellular oxidative stress, which is important for ATG4B oxidation and enhanced autophagy.^18^ In addition, as a response to the integrated stress response during mitochondrial oxidative stress, global translation may be inhibited by eIF-2*α*P via PKR (dsRNA-inducible protein kinase), whereas stress-specific gene expression is activated.^31^ Therefore, during chronic hyperglycemia and oxidative stress, excess mitophagy depletes ubiquitin adapters (e.g., p62/SQSTM1) either via lysosomal degradation and/or via reduced expression, resulting in the accumulation of ubiquitinated organelles in the cytosol. This process will result in cytoplasmic crowding and a loss of cytosolic sanctity, generating further ROS/RNS stress in a vicious cycle of oxidative stress, mitophagic flux and p62/SQSTM1 depletion. P62/SQSTM1 is also important for removal of misfolded and aggregated proteins by autophagy.

Our data support such a hypothesis in that TXNIP knockout restores LC3B (I and II) and p62/SQSTM1 protein levels under HG conditions and reduces mitophagic flux. Hence, unless the source of the ROS/RNS itself is eliminated or neutralized, excessive mitophagic flux will ultimately lead to the accumulation of ubiquitin-tagged damaged MT, which may be interpreted as a lack of mitophagy in chronic diseases. In fact, excess mitophagic flux will lead to autophagic cell death due to energy collapse. Therefore, regulating the intrinsic anti-oxidant capacity and normalizing ATG4B and p62/SQSTM1 and other ubiquitin adapter proteins will be important for maintaining a normal level of mitophagy flux and bioenergetics for cell survival in chronic nutrient access and neurodegenerative diseases. In addition, excess fragmentation of MT will lead to reduced ATP production, whereas ROS generation continues, thereby causing cellular injury and premature death.

Retinal neurons are known to be injured during the early stages of diabetes, and Müller cells react to retinal injury (gliosis), as indicated by radial GFAP expression. Photoreceptors in the ONL generate large amounts of ROS in the diabetic retina.^32^ During mitochondrial stress and depolarization, one potential route for mitophagy induction is that ATP synthesis is reduced and the AMP/ATP ratio increases.^33^ Subsequently, AMPK is activated and phosphorylates ULK1 (ATG1) and mTORC1.^34^ mTORC1 is an inhibitor of autophagy, and its phosphorylation by AMPK removes the inhibitory effect on autophagosome formation. Alternatively, we showed previously that sustained HG concentrations activate HIF-1*α* activity in rMC1 cells.^8^ HIF-1*α* induces BCL2/Adenovirus E1B 19 kDa interacting protein 3 expression and replaces the Bcl2-beclin 1 interaction.^35, 36^ The released beclin 1 also increases the autophagy flux. Furthermore, the role of PINK1 accumulation in the mitochondrial outer membrane under hyperglycemia and recruitment of Parkin as well as Drp1 activation need to be investigated further.^15, 16^

In summary, our results provide evidence for a critical role of TXNIP in mitophagy in retinal Müller glial cells under diabetic conditions and also potentially in early DR. During oxidative/nitrosative stress, the activation of the TXNIP–ATG4B–LC3BII and TXNIP–Drp1–Parkin–OPTN (p62/SQSTM1) axis may be one mechanism of enhanced mitophagy in retinal cells under HG conditions and in the diabetic retina, which will ultimately lead to the depletion of essential proteins and a decrease in mitochondrial number. Hence, TXNIP provides an excellent target for gene and drug therapy to prevent and slow the progression of DR. In addition, the CRISPR/Cas9 and TXNIP gRNA method employed in this study may also be a potential strategy for knocking out TXNIP and preventing DR progression, as well as other microvascular complications of diabetes.

## Materials and Methods

### Materials

DMEM (cat #10-014-CM) was purchased from Mediatech Inc. (Manassas, VA, USA) and Ham’s F12 was purchased from HyClone ((Logan, UT, USA) cat# SH30026.01), whereas serum (cat# MT35010CV) was purchased from Corning. Antibiotics and trypsin were purchased from HyClone. TXNIP antibodies were obtained from MBL and Santa Cruz Biotech (Dallas, TX, USA). A complete list of primary and secondary antibodies and their sources are shown in Supplementary Table S1. For immunofluorescence, prolong gold antifade reagent with DAPI (mounting medium, cat #P36935) was obtained from Molecular Probes (Eugene, OR, USA). Slides and coverslips were purchased from Fisher Scientific (Waltham, MA, USA).

### Methods

#### Cell culture

rMC1 cells were propagated in medium containing LG (1 g/l) DMEM/F12 (4 : 1 ratio), 5% fetal bovine serum, 100 U/ml of penicillin and 100 *μ*g/ml of streptomycin. After reaching ~70% confluence, the cells were maintained in 1% serum with either LG (5.5 mM) or HG (25 mM) for 5 days as previously described.^7^

#### Mitochondrial isolation

Cellular fractionation was performed using a slight modification of a previously used protocol.^37^ Briefly, cells were scraped and washed with PBS and resuspended in mitochondrial isolation buffer (3 mM HEPES–KOH (pH 7.4), 210 mM mannitol, 70 mM sucrose, 0.2 mM EGTA and protease inhibitor cocktail). Cells were then homogenized using a dounce homogenizer (~90 strokes). Cells were centrifuged at 2000 r.p.m. in an eppendorf 5417r centrifuge for 5 min to separate the nuclear fraction and the supernatant was then subjected to 5000 r.p.m. for 5 min. Pellet was discarded and the supernatant was centrifuged at 13 000 r.p.m. for 15 min at 4 °C to obtain MT in pellet. Pellet was washed twice with PBS and then re suspended in RIPA buffer containing protease inhibitors and sonicated briefly and spun at 2000 r.p.m. for 5 min. The supernatant was used for experiments. Protein was estimated by Bradford assay and 30 μg was used for western blotting.

#### MitoSOX assay

The formation of mitochondrial ROS in live rMC1 cells was detected by using the fluorescent probe MitoSOX Red (Molecular Probes, Manassas, VA, USA, M36008). This dye permeates MT, where it rapidly undergoes oxidation by superoxide, producing red fluorescence. The manufacturer’s protocol was used (Invitrogen, Carlsbad, CA, USA). Approximately, 5 × 10^3^cells per ml were cultured in 48-well plates, serum-starved overnight and exposed to glucose for the specified time period. Briefly, the cells were washed once with PBS and then incubated with MitoSOX (5 *μ*M) for 10 min at 37 °C. The cells were then washed thrice with PBS, and fluorescence was measured in a Gemini Fluorescent Microplate Reader (Molecular Devices, Sunnyvale, CA, USA) at Ex510/Em590 nm.

#### Mitochondrial membrane potential measurement by JC1

We used a JC1 dye (cat #T3168, Life Technologies, Carlsbad, CA, USA) to detect mitochondrial membrane depolarization in rMC1 cells after HG exposure for 5 days.^8^ JC1 penetrates the cell and accumulates within MT as orange-red aggregates, with Ex535/Em590 nm according to the manufacturer’s instructions.

#### CRISPR/Cas9 and TXNIP gRNA design and cleavage efficiency assay

Potential CRISPR/Cas9 gRNA targets containing the NGG protospacer adjacent motif^38, 39^ were designed to target EXON 1 of the rat TXNIP gene (ID:117514) and synthesized by Life Technologies. Of the three potential gRNA targets identified, TXNIP targets 1 and 3 were used in this study (Supplementary Figure S2).

#### Western blotting and QPCR

Western blotting (WB) was performed as previously described.^7, 8^ Briefly, proteins were extracted using RIPA buffer (Sigma, cat #R0278) containing protease/phosphatase inhibitors (Sigma, St. Louis, MO, USA, cat #MSSAFE). An amount of 30 *μ*g proteins was loaded for SDS-PAGE, and WB analysis was performed. ECL was used to detect the immunoreactive bands. Actin was used to normalize protein band intensities. Antibody sources and dilutions are shown in Supplementary Table S2. mRNA expression was analyzed by real-time reverse transcriptase PCR using the SYBR Green PCR Master Mix from Bio-Rad (Hercules, CA, USA) as previously described.^6, 7^ The PCR primers were purchased from Qiagen (Germantown, MD, USA) (Supplementary Table S2).

#### Immunofluorescence

The immunohistological methods for rMC1 cells were performed as described previously.^8^ Images of rMC1 cells were imaged on a Leica (Buffalo Grove, IL, USA) TCS S5P microscope, at a magnification of × 630 (oil), and the images were processed using Adobe Photoshop (Austin, TX, USA). Antibody sources and concentrations are shown in Supplementary Table S1.

### Diabetes induction in rats

Diabetes was induced in adult male Sprague–Dawley rats (∼275 g) via the injection of a single dose of STZ (65 mg/kg i.v.) dissolved in 0.01 M citrate buffer, pH 4.5, as described previously.^6, 7^ The rats were treated in accordance with the principles outlined in the NIH Guidelines for the Care and Use of Laboratory Animals and approved by the Institutional Animal Care and Use Committee. For TXNIP siRNA treatment, rat TXNIP mRNA siRNAs (#1330001, RSS332043) and negative control duplexes (#12935-300) were purchased from Invitrogen. We used a cell penetrating peptide MPG-δNSL to transfect the siRNAs as described previously.^6^

### Statistical analysis

The results are expressed as the mean±S.E.M. of the indicated number of experiments. Comparisons between two sets of experiments were analyzed using the unpaired two-tailed *t*-test, whereas one-way ANOVA followed by the Bonferroni *post hoc* test was used to determine differences among means in multiple sets of experiments. A *P*-value of <0.05 was considered to be statistically significant.
