# Supplementary material for: TXNIP regulates mitophagy in retinal Müller cells under high-glucose conditions: implications for diabetic retinopathy
Source: Cell Death Dis. 2017 May 11;8(5):e2777–. doi: 10.1038/cddis.2017.190 (PMC5520711; doi:10.1038/cddis.2017.190)
Supplement: Supplementary Information [file cddis2017190x1.pdf]

Supplementary data

Table S1. Primary and Secondary antibody sources

A. Primary Antibodies

Name	Animal source	Vendor	Cat #	Dilutions
1. TXNIP	mouse	MBL	KO205-2	1:2000 (WB)
2. TXNIP	mouse	Santa Cruz	sc-271238	1:200 (IF)
3. VDAC1	rabbit	Santa Cruz	sc-390996	1:200 (WB)
4. Th	rabbit	Thermo Scientific	PA5-17800	1:750 (WB)
5. mtHSP70	mouse	Thermo Scientific	MA1-094	1:2000 (WB)
6. GFAP	rabbit	Invitrogen	180063	1:200 (IF)
7. LC3B	rabbit	Thermo Scientific	PA1-46286	1:3000 (WB)
8. p62/SQSTM1	rabbit	Thermo Scientific	PA5-20839	1:1000 (WB)
9. COXIV	mouse	Life Technologies	A-21348	1:200 (IF)
10. LAMP2A	mouse	Thermo Scientific	MA5-17861	1:50 (IF)
11. LAMP2A	rabbit	Protein Tech	10397-1-AP	1:50 (IF)
12. Drp1	rabbit	Santa Cruz	sc-32898	1:50 (IF)
13. Parkin	rabbit	Santa Cruz	sc-83371	1:50 (IF)
14. Actin-β	goat	Santa Cruz	sc-1616	1:2000 (WB)
15. Ubiquitin	rabbit	Protein Tech	10201-AP	1:50 (IF)
16. Optineurin	mouse	Santa Cruz	sc-166576	1:50 (IF)

B. Secondary Antibodies:

Name	Vendor	Cat #	Dilutions
1. Anti-mouse 2°ry antibody HRP	Sigma	A5278	1:5000 (WB)
2. Anti-rabbit 2°ry antibody HRP	Sigma	A6154	1:5000 (WB)
3. Anti-goat 2°ry antibody HRP	Santa Cruz	sc-2020	1:5000 (WB)

4. Anti-mouse 2°ry Ab, Alexa Fluor 488	Jackson	115-545-146	1:400 (IF)
5. Anti-mouse 2°ry Ab, Alexa Fluor 594	Jackson	115-585-146	1:400 (IF)
6. Anti-rabbit 2°ry Ab, Alexa Fluor 488	Life Technologies	A-110 34	1:400 (IF)
7. Anti-rabbit 2°ry Ab, Alexa Fluor 594	Jackson	111-585-176	1:400 (IF)
8. Anti-goat 2°ry Ab, Alexa Fluor 488	Jackson	205-542-176	1:400 (IF)
9. Anti-goat 2°ry Ab, Alexa Fluor 594	Jackson	705-605-147	1:400 (IF)

Table S2. QPCR primer sets for rat

	Gene Name	Vendor (Qiagen)	Cat #
1.	TXNIP		QT00368984
2.	VDAC1		QT00190561
3.	Tyrosine hydroxylase, TH		QT00185024
4.	mtHSP70 (HSPa9)		QT01826419
5.	Aconitase 2		QT00187495
6.	Actin, beta		QT00193473
7.	TXNIP		PPR42837A
8.	ATG4B		PPR44299A
9.	Dynamin-related protein 1 (Drp1)		PPR44805B
10.	Fission 1		PPR42458A
11.	Mfn2		PRRR44137A
12.	Autophagy related ATG5		PPR50040B
13.	Parkin		PPR50238F
14.	Actin, beta		PPR06570C

Legend for Supplementary Figures:

Figure 1S. Purity of isolated mitochondrial fractions. Cytosolic, nuclear and mitochondrial fractions were isolated and tested on western blots the presence of cytosolic protein LDH (lactate dehydrogenase, ThermoFisher cat# PA5-27406) and nuclear protein lamin B1 (ProteinTech, cat# 66095-1-Ig) and mitochondrial outer membrane protein TOM20 (ProteinTech, cat# 11802-1-AP). As shown in Figure 1S, A (upper panel) and B (densitometric analysis), LDH is detected in the cytosolic fraction but not in nuclear and mitochondria fractions both in LG and HG conditions. Similarly, the nuclear protein lamin B1 is seen in the nuclear fraction, but not in the cytosol and mitochondria (Figure 1S, A, middle panel and C). Similarly, mitochondrial protein TOM20 is detected in the mitochondrial fraction under LG, while under HG, TOM20 expression in the cytosol appears to be increased. This may be due to mitochondrial dysfunction under HG and reduce cytosol to mitochondrial transport. The absence of cytosolic and nuclear proteins in mitochondrial fractions indicates purity of the isolated mitochondrial fractions. A representative of n=3 is shown in Figure A.

Figure 2S. **TXNIP levels are elevated in the mitochondria of rMC1 under HG. (A)** Co-localization analysis of mitochondria and TXNIP. rMC1 cells were stained for mitochondrial protein thioredoxin 2 (Trx2, red) and TXNIP (green) after 5 days of HG. There is co-localization of Trx2 and TXNIP under HG when compared with LG. Nuclei are stained with DAPI (blue). Scale bar represents 5 μm. A representative of n=2. **(B)** Upregulation of TXNIP by HG in rMC1 is not due to osmolar effect as 20 mM L-Glc+5 mM LG, which enters the cell but does not metabolize, has no significant effect on TXNIP expression (p=0.13, n=4). Similarly, we did not observe expression of TXNIP in immunofluorescence or co-staining with Trx2 with L-Glc (not shown).

Figure 3S. Mitophagy induction by mito-targeted drugs. In order to demonstrate mitophagy induction in rMC1 cells as controls, rMC1 cells were maintained under LG or HG for 24 h and different concentrations of CCCP (Sigma cat# C2759, Carbonyl cyanide 3-chlorophenylhydrazone, an ionophore which induces mitochondrial membrane depolarization and mitophagy) were added for 6h. (A) First, mitochondrial superoxide generation was detected

by mitosox. CCCP (10-40 μM) induces ROS generation in rMC1 cells both in LG and HG conditions. (B) Furthermore, using 20 μM CCCP and 0.1 μM Baf A (Sigma cat# SML1661-.1ML, Bafilomycin A1, inhibiting vacuolar H+ ATPase and lysosomal function) we observed on western blots increased levels of LC3BII, p62, and Optineurin (OPTN) in the presence of BafA suggesting mitophagy flux to lysosome. Actin is not changed by CCCP or BafA. Densitometric quantitation of the bands is shown in Figure 3S-E (separate Figure). (C) Figure shows a diagram of the action of CCCP and BafA in mitophagy. (D) Furthermore, CCCP induces co-localization of the mitochondrial complex IV protein (COXIV) and lysosomal Lamp2A indicating mitophagy. On the other hand, when BafA is present, autophagosome and lysosome fusion is prevented, which is indicated by the presence of separate COXIV and Lamp2A membrane vesicles (arrows).

Figure 3S-E. Densitometric analysis of the western blots in Figure 3S-B. In the presence of Baf-A, LC3BII, p62 and OPTN are significantly enhanced when normalized to Actin. LC3BI is not significantly altered by CCCP with or without BafA.

Figure 4S. CRISPR/Cas9 and TXNIP gRNA design and cleavage efficiency assay. (A-B) Three potential gRNA targets were identified in rat TXNIP EXON1 (gene ID:117514) and **(C)** (i) TXNIP gRNA targets 1 and 3 were synthesized via *in vitro* transcription (IVT). Isolated RNAs were run on gel electrophoresis and observe that both run as a single band as expected at ~100 bp for a gRNA. Both gRNAs TXNIP1 and TXNIP3 together were transfected with Cas9 mRNA in rMC1 cells and analyzed further for gene knockout. (ii) In order to assess the cleavage efficiency, the GeneArt Genome Cleavage kit was used and samples were run on a 2% agarose gel. **(a)** Control Template panel shows a positive reaction of the endonuclease provided by the manufacturer. In T(1+2) cells (two different cell lines), three DNA fragments of sizes ~270, 170 and 75 bp together with the original fragment were obtained and densitometric analysis was performed. The DNA marker used here is O'RangeRuler 50 bp DNA Ladder (Thermo Scienfitic, Cat #SM0613). **(b)** There was no gDNA cleavage in control rMC1 cells, indicating specificity of the gRNA treatment. **(iii)** The cleavage efficiency was calculated as 42.5% using the formula: Cleavage Efficiency = 1-[(1-fraction cleaved) 1/2)] where fraction cleaved is the sum of total cleaved band intensities/sum of the cleaved and parental band intensities. **(D)** The protein level

for TXNIP in rMC1 on western blots after CRISPR/Cas9 and TXNIP gRNA editing was significantly decreased (p=0.00033, n=4) both in LG and HG when compared with the control rMC1 cells and **(E)** by immunohistochemistry (TXNIP, green, nuclei-blue, DAPI).

Figure 5S. Ubiquitin receptor p62/SQSTM1 is also associated with the mitochondrial protein COXIV in HG suggesting its possible role in mitophagy. (A) We observed that the level of p62/SQSTM1 is significantly reduced (p=0.03, n=4) under HG glucose when compared to LG, suggesting its degradation via the lysosomal pathway. Indeed, bafilomycin A, which prevents autophagosome fusion with the lysosome, also restores p62/SQSTM1 level under HG, confirming p62 degradation via the lysosomal pathway (not shown). **(B)** Furthermore, after TXNIP knockout, p62/SQSTM1 level is restored to the level of LG (p=ns, n=4). **(C)** p62/SQSTM1 colocalizes with mitochondrial COXIV under HG in rMC1 (upper two panels, inset, arrow), which is also prevented by TXNIP knockout (lower two panels).

Figure 6S. High-glucose medium regulates autophagic ATG4B under high glucose in rMC1 cells. (A) Compared to LG medium, the treatment of rMC1 cells with HG medium for 5 days induces a significant reduction of ATG4B mRNA (p=0.001; n=4). **(B)** Furthermore, less staining of ATG4B (red) is observed in rMC1 cells than in T1+3 cells under HG condition. Nuclei stain blue with DAPI. Conversely, ATG4B staining is higher in T(1+3) cells both under LG and HG when compared to rMC1 cells. A representative of n= (3) is shown for all images.

Figure 7S. TXNIP expression is increased in the diabetic rat retina and mediates LC3BII-autophagosome formation. (A) TXNIP mRNA **and (B, C)** protein levels are significantly increased in diabetic rat retinas, as indicated by QPCR (p<0.007 and n=5) and WB (p=0.009; n=4), when compared with non-diabetic rat retinas. **(D)** IHC also shown increased TXNIP staining in the diabetic retina compared to normal rat retina and intravitreal injection of siRNA to TXNIP reduces TXNIP expression. **(E)** LC3BII puncta are increased in the diabetic retina than in the normal rat retina, which is also reduced by siTXNIP. Inset shows magnification of the puncta. **(F)** Further, the induction of radial GFAP, an indicator of Muller glia activation, gliosis, is also seen in the diabetic retina compared to the normal retina, which is prevented by siTXNIP. The results suggest that TXNIP is involved in autophagic and/or mitophagic flux in the diabetic

retina and as well as glial activation. Further studies are undertaking to ascertain the role of TXNIP in mitophagy in diabetic retinopathy.

Figure 8S. Diabetes induces alterations in mitochondrial and neuronal gene expression in the diabetic rat retina. Mitochondrial VDAC1 expression is elevated both at the **(A)** mRNA ($p<0.05$, n=6) and **(B)** protein levels ($p<0.05$, n=4). **(C)** The mRNA level of Kreb cycle enzyme aconitase 2 (Aco2) is also increased in diabetes in the retina ($p<0.03$, n=6), suggesting mitochondrial oxidative stress. Aco2 expression is considered as a response to mitochondrial matrix oxidative stress as the enzyme has iron-sulfur complexes that are exposed to the exterior, and frequently attached by ROS. **(D)** In agreement, heme-oxygenase 1 (HO-1) expression is induced. Furthermore, the expression of both **(E)** tyrosine hydroxylase (Th) and **(F)** synaptopodin (dendritic protein) are also significantly reduced indicating neuronal (dopaminergic neuron) injury. **(G)** Autophagic gene expression for ATG5 is upregulated while **(H)** mitochondria targeted heat-shock protein/chaperone, mtHSP70, expression remains unchanged. These data further suggest mitochondrial dysfunction and mitophagic signal activation in diabetic rat retinas when compared to normal rat retinas.

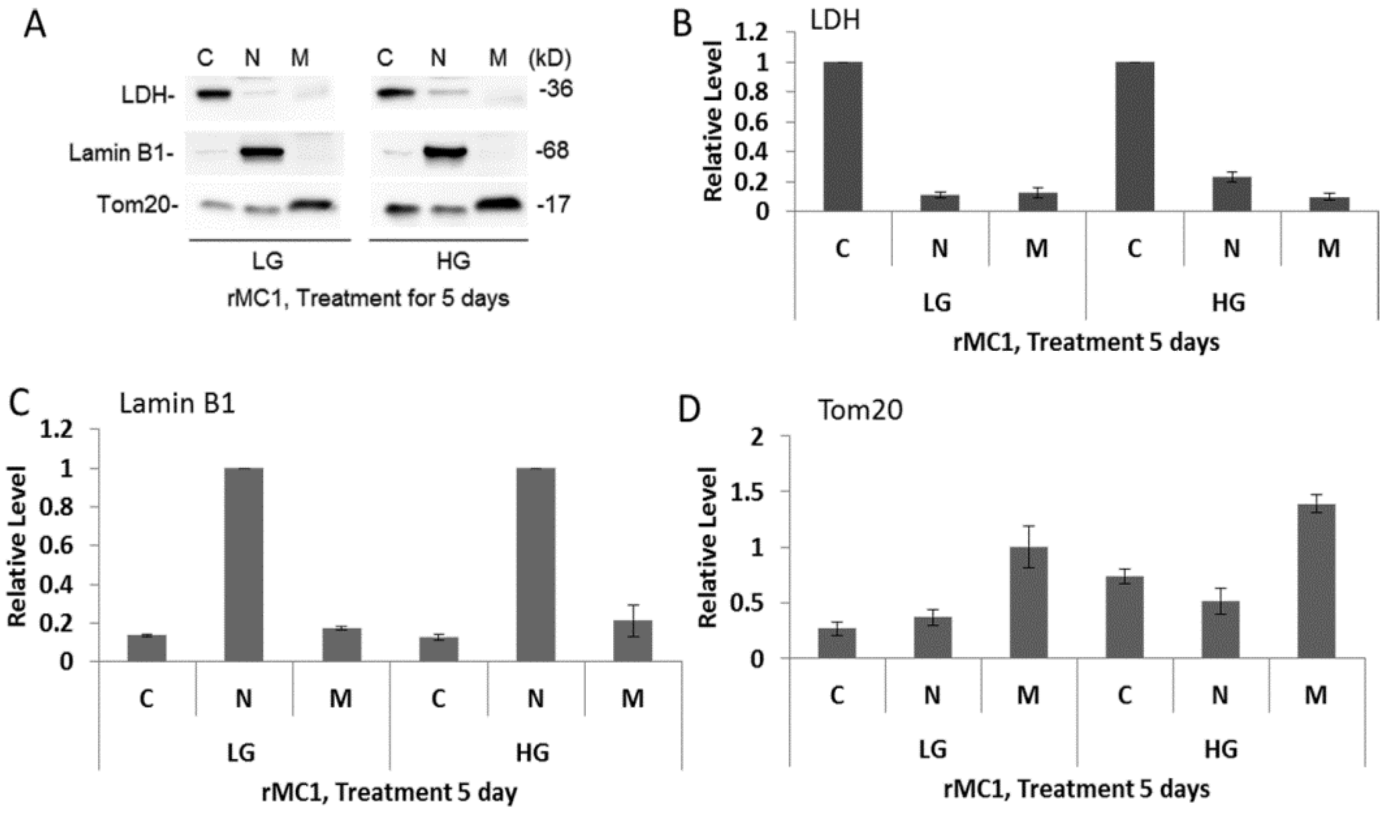

Figure 1S. Purity of isolated mitochondrial fractions.

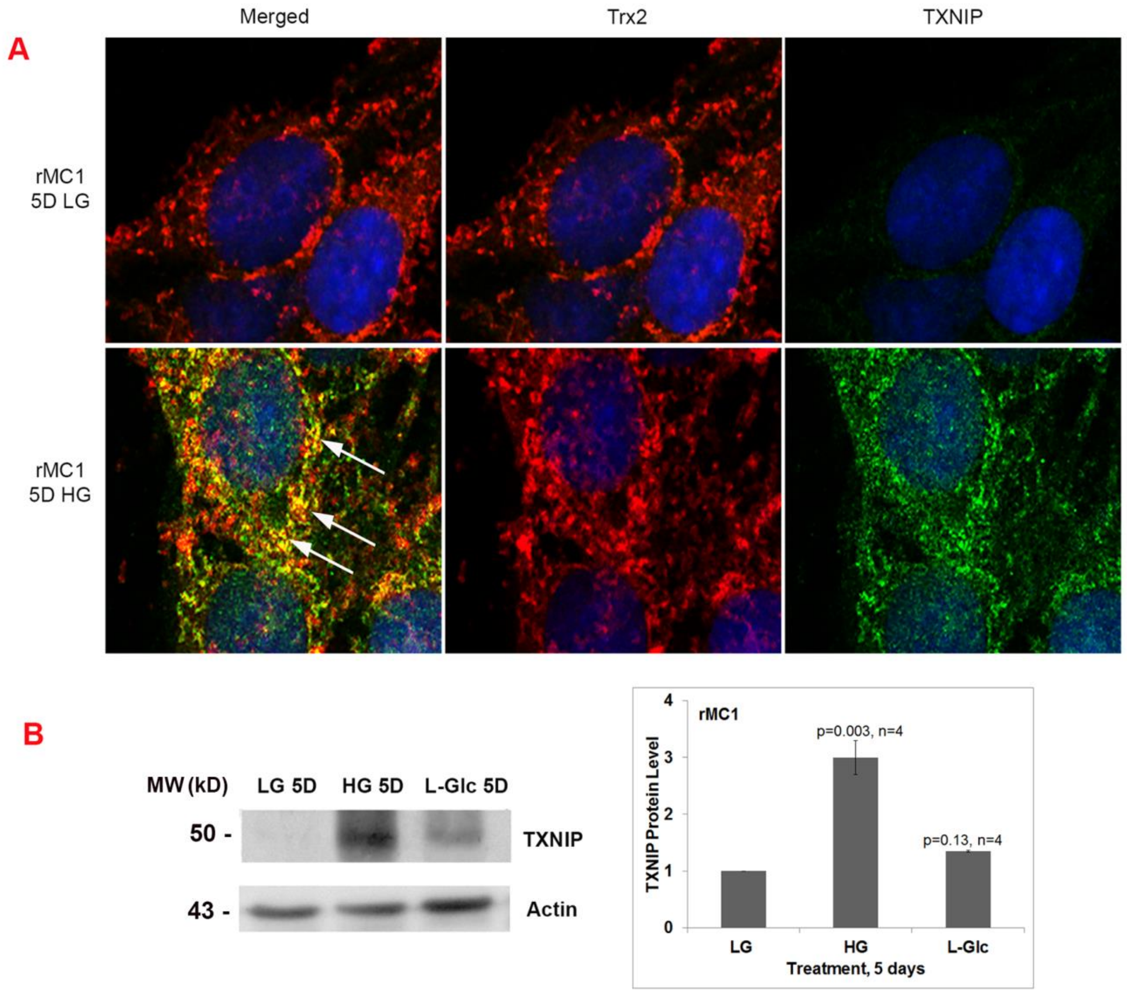

Figure 2S. TXNIP levels are elevated in the mitochondria of rMC1 under HG.

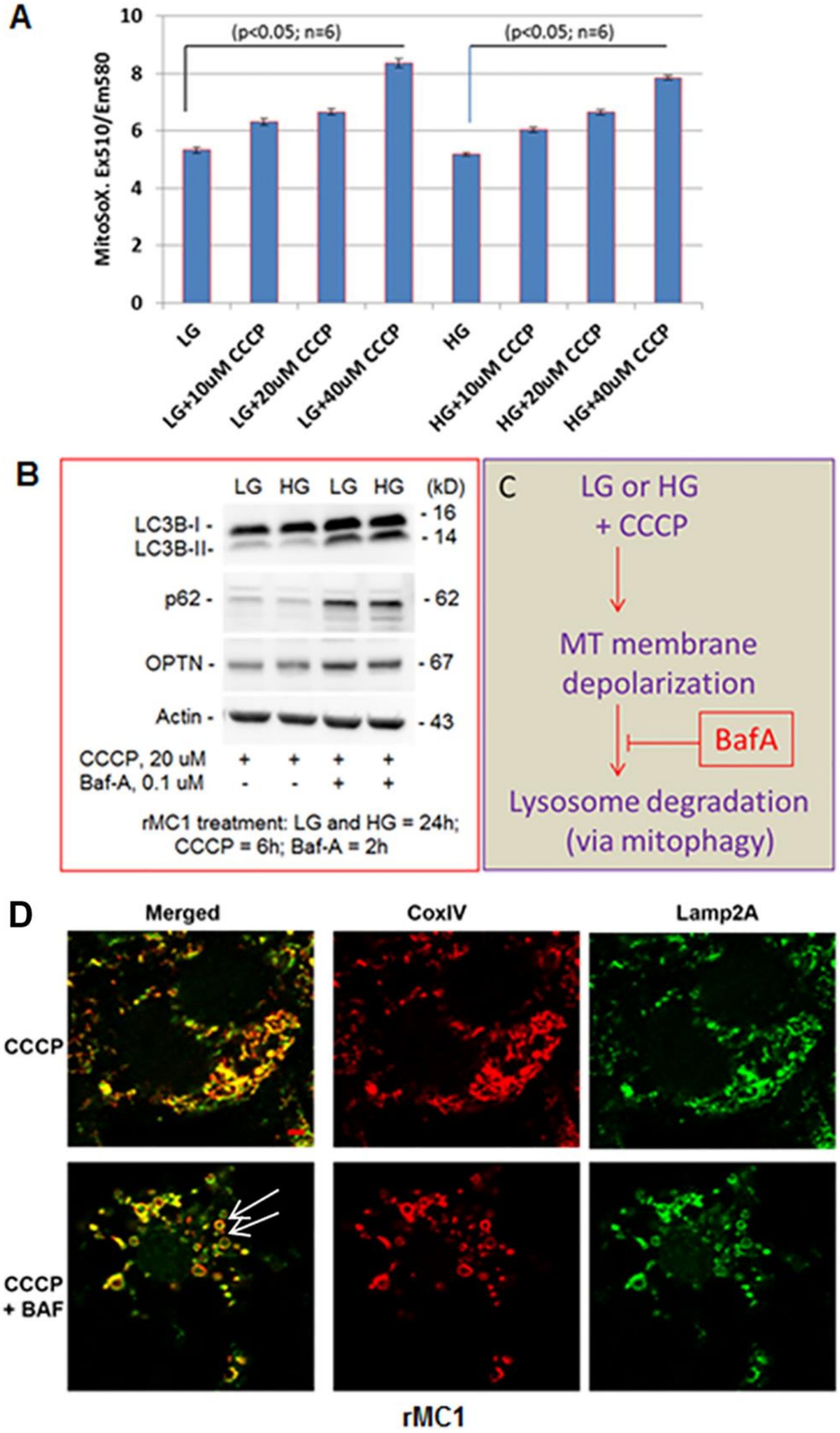

Figure 3S. Mitophagy induction by mito-targeted drugs.

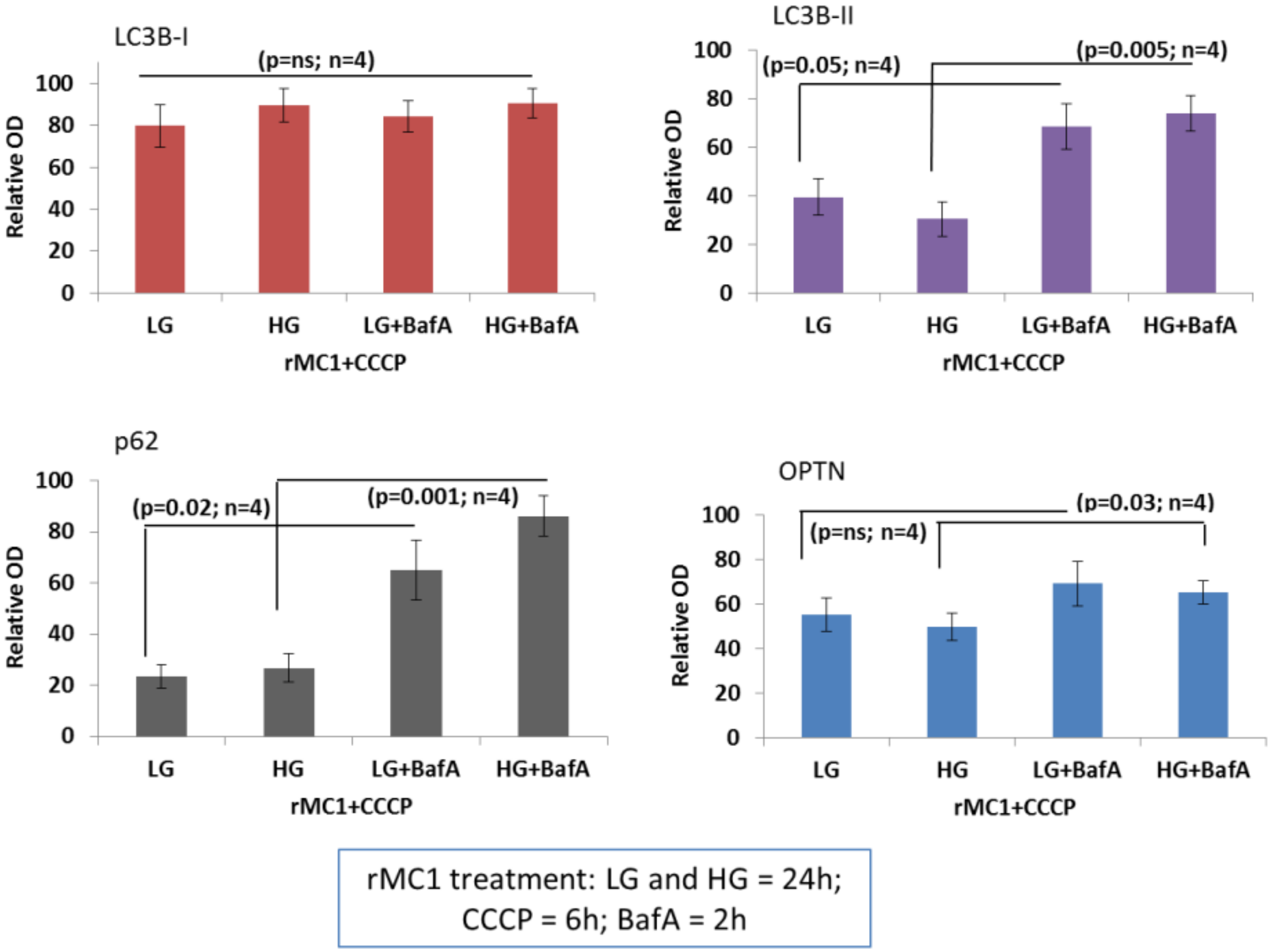

Figure 3S-E. Densitometric analysis of the western blots in Figure 3S-B.

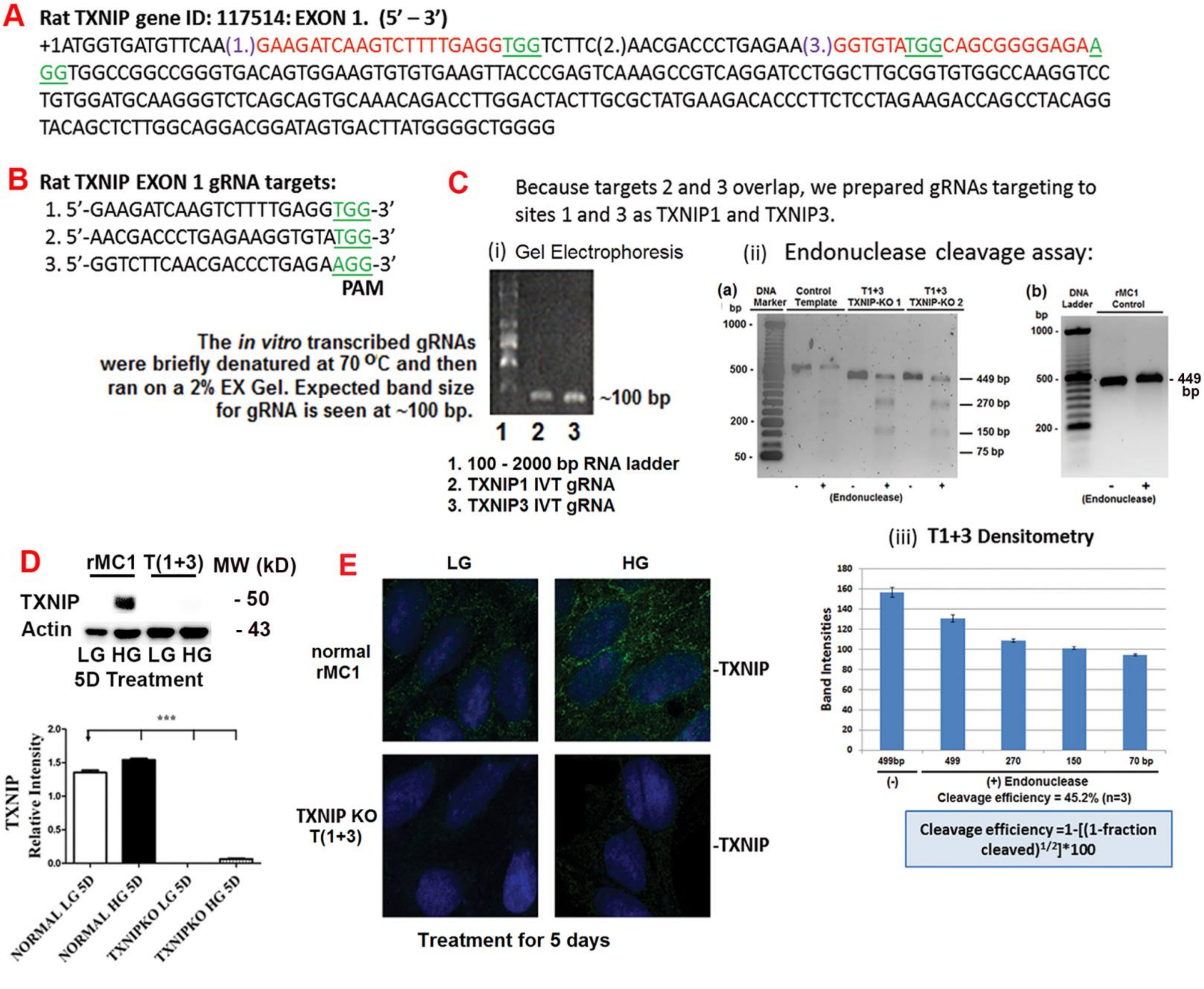

Figure 4S. CRISPR/Cas9 and TXNIP gRNA design and cleavage efficiency assay.

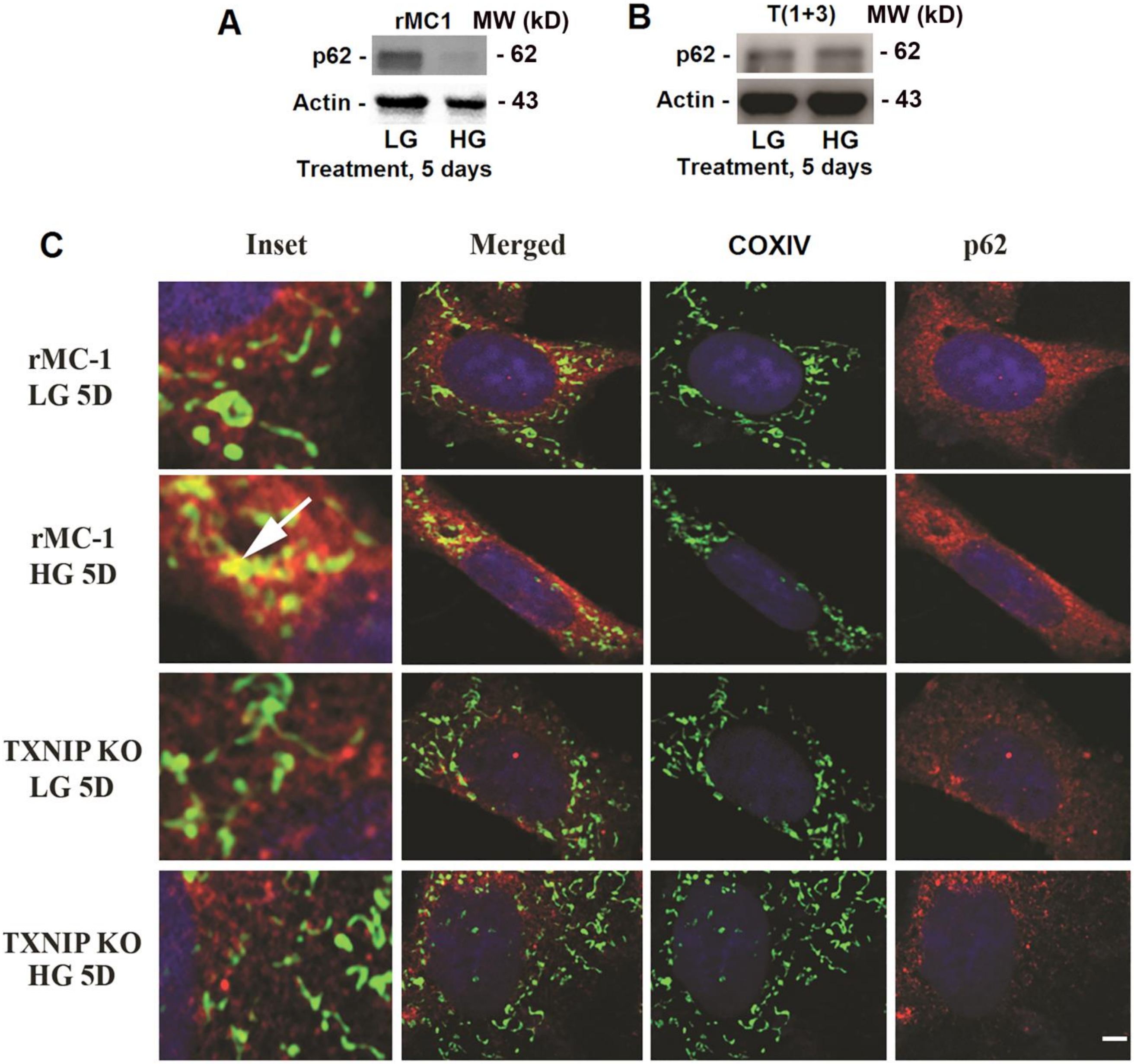

Figure 5S. Ubiquitin receptor p62/SQSTM1 is also associated with the mitochondrial protein COXIV in HG suggesting its possible role in mitophagy.

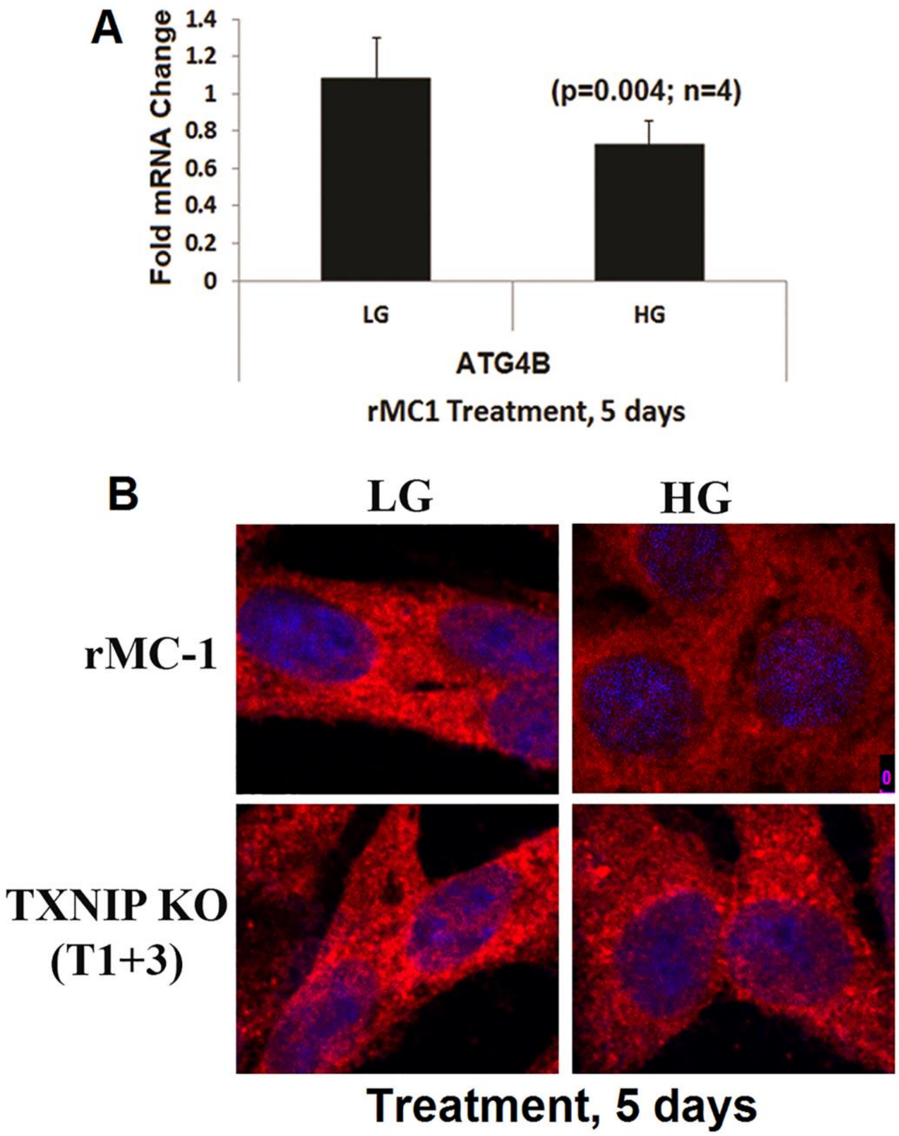

Figure 6S. High-glucose medium regulates autophagic ATG4B under high glucose in rMC1 cells.

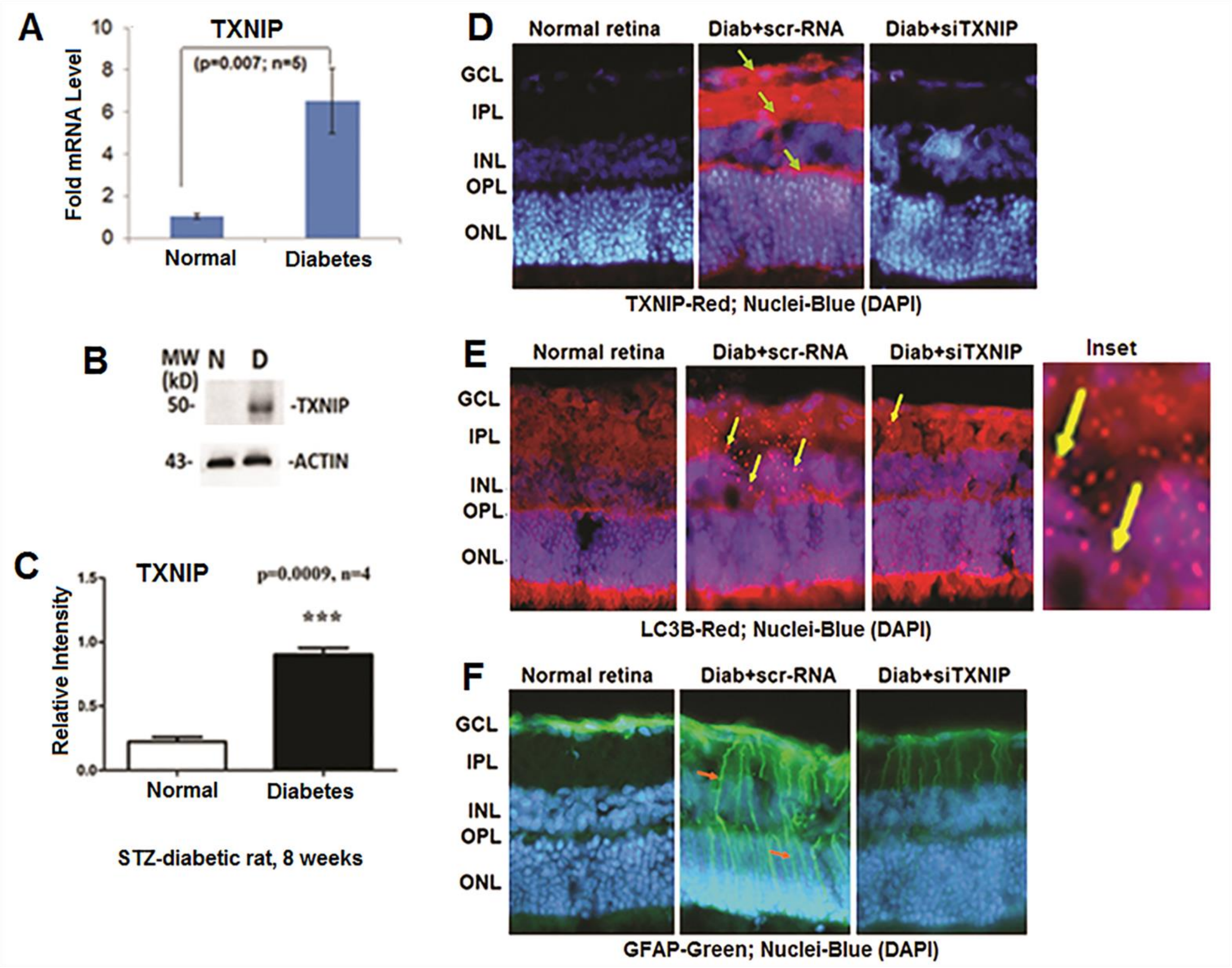

Figure 7S. TXNIP expression is incred in the diabetic rat retina and mediates LC3BII-autophagosome formation.

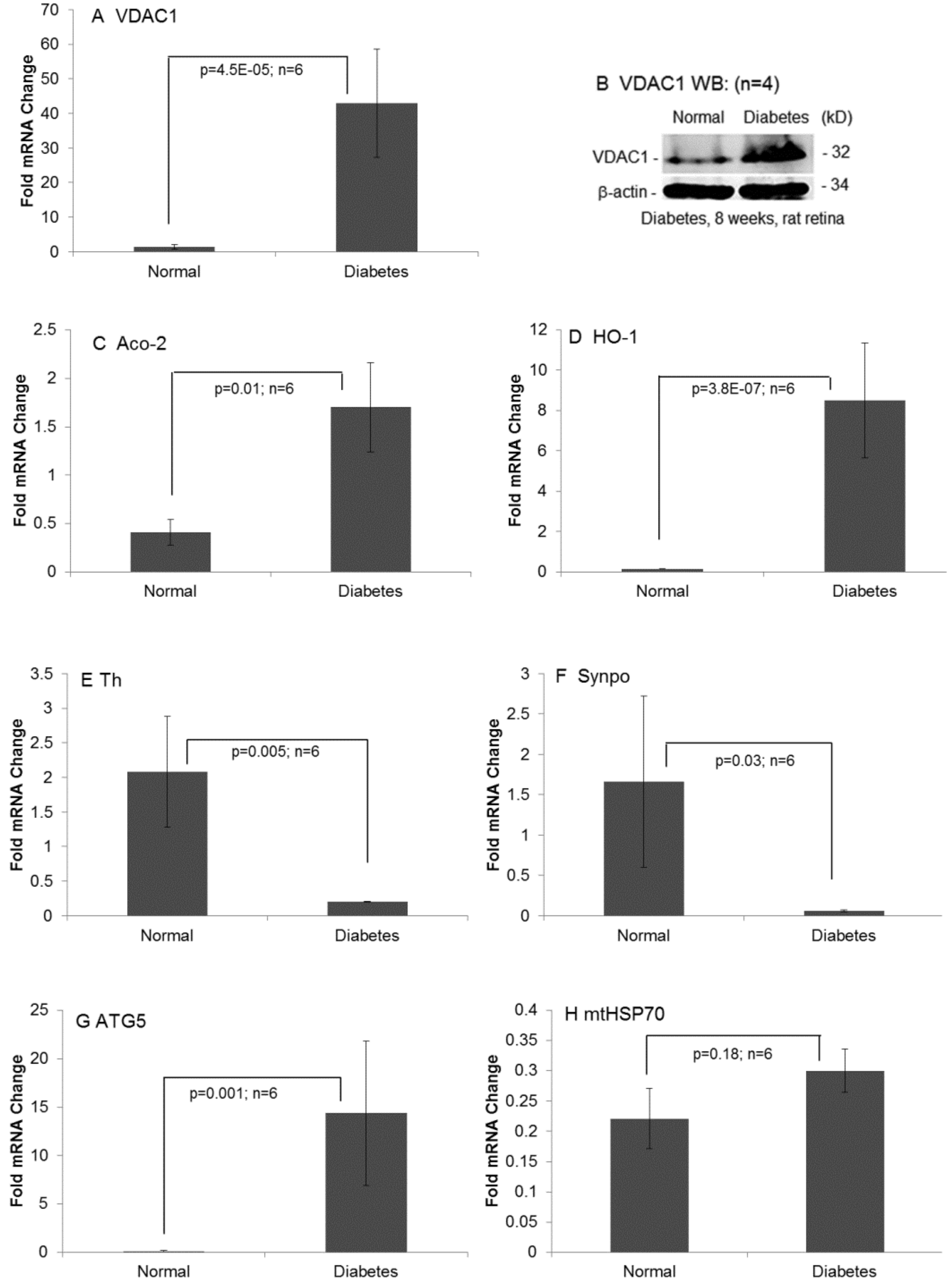

Figure 8S. Diabetes induces alterations in mitochondrial and neuronal gene expression in the diabetic rat retina.
